# Importance of tissue sampling, laboratory methods, and patient characteristics for detection of *Pneumocystis* in autopsied lungs of non-immunosuppressed individuals

**DOI:** 10.1007/s10096-017-3006-8

**Published:** 2017-06-05

**Authors:** S. L. Vargas, C. Ponce, R. Bustamante, E. Calderón, G. Nevez, Y. De Armas, O. Matos, R. F. Miller, M. J. Gallo

**Affiliations:** 1 Programa de Microbiología y Micología, Instituto de Ciencias Biomédicas, Facultad de Medicina, Universidad de Chile, Independencia 1027, 8380453 Santiago, Chile; 2 Centro de Investigación Biomédica en Red de Epidemiología y Salud Pública (CIBERESP) and Instituto de Biomedicina de Sevilla, Hospital Universitario Virgen del Rocío/CSIC/Universidad de Sevilla, Seville, Spain; 3 Laboratory of Parasitology and Mycology, Brest University Hospital, & University of Brest, GEIHP, EA 3142 Brest, France; 4 Hospital Microbiology Department, Institute of Tropical Medicine “Pedro Kourí” Pathology Department, Institute of Tropical Medicine “Pedro Kourí” Hospital, Havana, Cuba; 5 Unidade de Parasitología Médica, Grupo de Protozoários Oportunistas/VIH e Outros Protozoarios, Global Health and Tropical Medicine, Instituto de Higiene e Medicina Tropical, Universidade NOVA de Lisboa, 1349-008 Lisbon, Portugal; 6 Research Department of Infection and Population Health, Institute of Global Health, University College London, Mortimer Market Street, London, WC1E 6BT UK; 7 Clinical Research Department, London School of Hygiene and Tropical Medicine, London, UK; 8 Servicio Médico Legal, Av. La Paz 1012, 8380454 Santiago, Chile

## Abstract

To understand the epidemiological significance of Pneumocystis detection in a lung tissue sample of non-immunosuppressed individuals, we examined sampling procedures, laboratory methodology, and patient characteristics of autopsy series reported in the literature. Number of tissue specimens, DNA-extraction procedures, age and underlying diagnosis highly influence yield and are critical to understand yield differences of Pneumocystis among reports of pulmonary colonization in immunocompetent individuals.

## Introduction

It is well-known that the fungus *Pneumocystis jirovecii* displays tropism for the lungs and causes severe pneumonia in patients immunocompromised by HIV infection or other causes of immunosuppression [1]. However, in the broad context, *Pneumocystis* pneumonia (PCP) is an exceptional event within the general population; while it is increasingly evident *P. jirovecii* colonization is of widespread occurrence and points to the need to understand significance of this organism beyond the pneumonia [2–4]. The most common significance of this mild infection, termed “colonization”, is the human-to-human transmission of the fungus, meaning immunocompetent individuals are likely participants in the circulation of this pathogen in the community [2, 5, 6]. Transmission studies can be done using non-invasive respiratory samples. However, beyond transmission studies, *Pneumocystis* is no longer considered a commensal, and clarification of any pathogenic role of *P. jirovecii* in the causation of lung disease in immunocompetent hosts would greatly benefit from a clearer understanding of the epidemiology of, and tissue distribution of *Pneumocystis* in human lung specimens. A pathogenic role is suggested by the association of *P. jirovecii* and increased severity of chronic obstructive pulmonary disease (COPD), by the detection of increased mucus associated with *P. jirovecii* in the lungs of infants dying unexpectedly in the community, and by the recently described link between *P. jirovecii* colonization and asthma [6–8]. Detection of *Pneumocystis* in human lungs is difficult as the organism will not grow in microbial culture, adopts a characteristically focal distribution in the lungs, and requires a specific search using DNA amplification techniques and/or specific stains that need trained experienced microscopists to identify the cystic and the more abundant trophic biological forms. The high prevalence of *P. jirovecii* within the general population, and new associations with human disease that are being described, underscore the importance of optimizing tissue sampling and standardizing diagnostic methodology, which are needed to recognize the epidemiology and to understand any role of this fungal organism in lung disease.

A recent publication describing the prevalence of *P. jirovecii* colonization in lungs of the general population in Turkey [3], using DNA amplification techniques, confirms previous reports detecting *Pneumocystis* in the autopsied lungs of immunocompetent individuals from the general population dying in the community in Chile [2, 5] and in the United States [4], and motivated us to compare the sampling and diagnostic processes underlying the different yields in *Pneumocystis* detection between these studies (Table 1). Understanding detection methodology is pivotal to compare epidemiology between countries, and will lead to improved diagnostic methodologies, to reliable collaborative studies, and ultimately to reach a better understanding of disease.

**Table 1:** Detection of *P. jirovecii* DNA from autopsy lung tissue of immune competent adults and infants

Reference	Geographical location		
	Turkey	Chile	United States
	Ref. [3]	Ref. [2]	Ref. [4]
Patient group	No PCR positives for *P.jirovecii* DNA/No sampled (%)		
Adults with cause of death due to violence, suicide, or accident	13/111 (12)	34/55 (62)	NA
Adults with cause of death due to an underlying medical condition	20/73^a^ (27)	15/19^b^ (79)	NA
Infants, age < 12 months	5/11 (43)	105/128 (82)	58/58 (100)

*PCR* polymerase chain reaction, *NA* not applicable

^a^Diagnoses: cardiovascular conditions including myocardial infarction (*n* = 48), pulmonary infection, edema (*n* = 14), subarachnoid hemorrhage (*n* = 7), pericardial tamponade (*n* = 2), epileptic seizures (*n* = 1), and intestinal obstruction (*n* = 1)

^b^Diagnoses: myocardial infarction (*n* = 13), cardiac tamponade (*n* = 1), stroke (*n* = 1), subarachnoid hemorrhage (*n* = 1), digestive hemorrhage (*n* = 1), peritonitis (*n* = 1), and intestinal obstruction (*n* = 1)

## Materials and methods

### Lung sampling technique

The Chilean series examined between 2 and 15 (median 7) lung tissue samples, each weighing 0.5 g, representing up to 3% of the weight of the right upper lobe. *Pneumocystis*-negative samples were confirmed as negative after analysis of 7% of lung weight. Magnetic agitation with individually sterilized magnets was used to homogenize samples [2, 6]. Samples were centrifuged at 2900 g for 10 min. Özkoç et al. [3] analyzed a single 1 g sample from the right upper lobe of each patient. These were mechanically homogenized, and centrifuged at a speed of 690 g for 10 min. Beard et al. [4] obtained four samples (two from each upper lobe) and centrifuged at 14000 g for 5–7 min. The influence of the lower centrifugation force used by Özkoç et al. on their lower yield for detecting *Pneumocystis* DNA is difficult to interpret. However, forces over 1000 g are generally used.

**Fig. 1:**
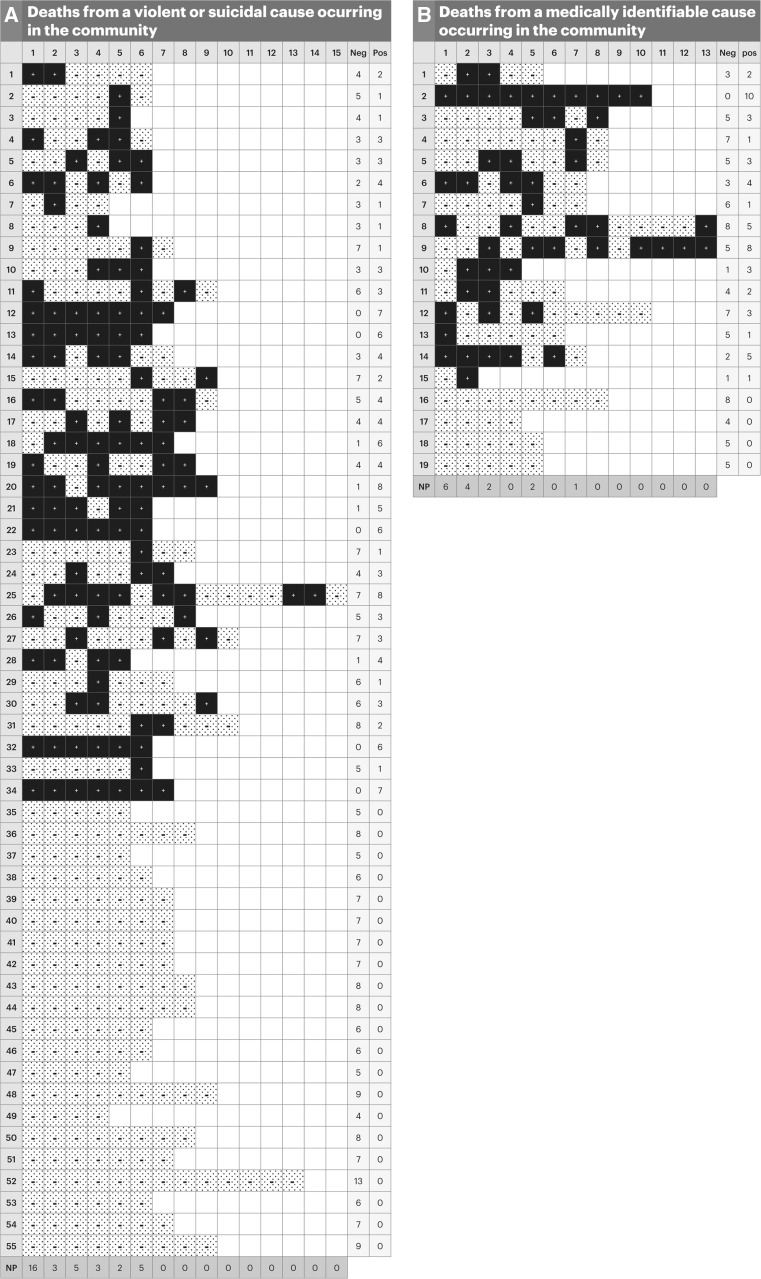
Detection of *P. jirovecii* DNA in autopsy lungs from adults, using n-PCR from multiple lung samples obtained from the right upper lobe. *Horizontal axis* shows the number of 0.4 g lung samples analyzed. *Vertical axis* numbers represent individual adults. **a** Fifty-five adults dying in the community from violence, accident or suicide. **b** Nineteen adults dying in the community from an underlying medical cause (expanded from Ref. [2]). *Positive image* positive for Pneumocystis-DNA, *Negative image* Negative for Pneumocystis-DNA, *NP* New positive individuals that were detected after the analysis of the 1st, 2nd, 3rd, ...etc. lung sample. This shows that in adults from the general population dying in the community there were no new positives or changes in detection results after the analysis of seven samples

When just the first of all lung samples was analyzed, results in Chilean samples show that *Pneumocystis* DNA was detected in 16 (29%) of 55 adults with violent deaths, and in 6 (31.5%) of 19 adults dying from medical conditions (Fig. 1). This yield for detection of *Pneumocystis* from just the first analyzed lung sample is still higher when compared to the 12% and 27% reported by Özkoç et al. in samples from Turkey for violent deaths or for deaths from medical conditions, respectively (Table 1). Data from Beard et al. is not available regarding the yield from a single or more than one lung sample in the US study.

### Method used to extract DNA

Vargas et al. used the QIAmp DNA extraction kit (QIAGEN), Özkoç et al. used the Machery-Nagel extraction kit, and Beard et al. used the Wizard Genomic DNA purification kit (Promega). It is not known how these DNA extraction processes perform relative to the QIAmp kit. Huggett et al. showed that the amount of *P. jirovecii* DNA extracted from BAL fluid samples was dependant on which extraction kit was used; the yield using QIAmp was higher than that using DNAeasy [9]. QIAmp recovered more DNA than the Promega kit for specifically detecting *Mycobacterium leprae* on tissue samples [10]. In none of the three studies that are compared were extracted DNA yield or DNA quality parameters documented using A260/280 (yield purity) or A260/230 (quality/contamination) ratios. Amplification reactions in the Chilean series were run using human β-globin as an internal control to monitor for DNA-inhibition and liver tissue as a *P. jirovecii*-negative control to monitor for cross contamination. However, no assessments were made to monitor for DNA yield or quality parameters in any of the manuscripts reviewed in this article. Promega, Quiagen, and Macherey-Nagel DNA extraction kits yield amounts of DNA that may vary depending on elusion volumes according to their manufacturer manuals. Interestingly, comparisons of the DNA amount recovered from the same bronchoalveolar lavage samples from immunocompromised patients extracted by these kits as done by Huguet et al. using DNA-easy and QIAamp favored the QIAamp kit over the DNA-easy kit (12.5-fold yield versus 2-fold yield) [9]. The potential impact of these differences in diagnostic sensitivity is unknown and may reflect the burden of *Pneumocystis* in the sample. Whether detection of a very low and focal burden of *Pneumocystis* organisms, as in lungs from immunocompetent adults, is improved using a kit that performs favorably in extracting a higher amount DNA, as indicated in the manufacturer manuals, remains to be determined. We have compared Promega and Quiagen kits in the same nasopharyngeal aspirate samples from immunocompetent infants that have a higher *Pneumocystis* burden than adults, and, although DNA was not measured, diagnostic results were consistent for nested-PCR in these infants (Ponce, CA; unpublished).

### DNA amplification protocol

Studies from Chile, Turkey, and the United States amplified DNA using the multi-copy gene mtLSUrRNA. Consideration to quantitative PCR protocols should be given in future studies as they will provide a better estimate of the amount of *P. jirovecii* DNA in a given sample. However, the epidemiological and diagnostic relevance of *P. jirovecii* quantitation will need to be documented because a given pulmonary burden of organisms may have a different meaning in different patient contexts. For example, non-HIV patients with PCP have paradoxically a lower burden of *P. jirovecii* organisms and more severe pneumonia than patients with HIV-related PCP, therefore indicating that *Pneumocystis* disease is host dependent and disease severity does not correlate with *Pneumocystis* burden. *P. jirovecii* colonization of undetermined burden has been associated with old age, or increased severity of bacterial pneumonia in non-immunosupressed patients [11]. A major difficulty in interpreting quantitative PCR is the similar burden levels that can overlap in patients that are colonized and in patients with PCP [12].

### Age of infants studied

Among infants, Vargas et al. [5, 6, 13, 14] and Larsen et al. [15] have previously shown that detection rates for *P. jirovecii* vary by age; with a peak at 2–5 months of age and declining thereafter. In the study by Özkoç et al., the ages of infants <12 months of age are not given, but two of 11 infants died 1–2 days after birth, thus inferring that they would be unlikely to have had time to become heavily colonized with *P. jirovecii*.

### Mode or diagnosis of death

Although a decreased CD4+ T-cell lymphocyte count is the strongest predictor for *Pneumocystis* susceptibility, respiratory disease appears to influence the detection rate of *Pneumocystis* among the immunocompetent population in these studies. Respiratory disease was diagnosed only in the series from Turkey, where individuals dying from medical conditions had significantly increased detection of *P. jirovecii* DNA than those dying from other causes. Immunosuppressive illnesses were not diagnosed [2, 3].

## Discussion

### Amount of lung tissue and tissue distribution

The smaller amount of lung tissue examined may partly explain the lower yield reported by Özkoç et al. when compared to results from Chile or from the United States. However, previous publications also identify distribution of *P. jirovecii* in lungs is heterogeneous [16, 17], and a study of immunocompetent patients with COPD shows that *Pneumocystis* may be more abundant in the lower lobes of the lung [7]. The focal distribution of *Pneumocystis* determines the need to sample more than one site to detect colonization. Importantly, in the Chilean series in adults, no new positives were detected after seven samples were analyzed (Fig. 1).

Studies in infants from Chile analyzed up to six samples in infants totaling the same 3% of lung tissue weight as in adults. These studies used a 0.2-g tissue sample per lung lobe sampling the three right lung lobes. Those infants having at least one positive sample were considered positive, and negative infants were sampled a second time for confirmation obtaining 0.4 g from the right upper lobe. A total of 20% of *Pneumocystis*-negative infants on first sample changed to positive after analysis of the second sample, providing a final *Pneumocystis* detection rate in infants of 82% [6]. Of interest, inter-lobe consistency between the three lobes was 59% when only first sampling analysis was considered, suggesting all lobes need to be studied [6]. A more recent series analyzing one, two, or three individual lung tissue samples of 0.4 g each adding up to 3% of the upper right lung lobe weight give consistent nested-PCR detection results for each sample, and negative results do not change after analysis of additional tissue (Ponce et al., unpublished). Inter-lobar consistency is being evaluated.

Therefore, the *Pneumocystis* detection yields in autopsy samples from Turkey, and from both Chile and the United States need to be interpreted in the perspective of the number of samples analyzed. They confirm that the prevalence of *P. jirovecii* in autopsy lung material from immunocompetent adults is high, focal in distribution, and with a very low burden of *Pneumocystis* organisms as evidenced by immunofluorescence microscopy examination in the Chilean series [2], and by the negative results of a previous study using a less sensitive, single-round PCR technique in post-mortem lungs from non-immunosuppressed individuals 15 to 70 years of age from the United Kingdom [18].

The lower burden of pulmonary colonization by *Pneumocystis* in non-immunosuppressed adult individuals makes it unlikely that non-invasive sampling will replace the study of lung specimens for detection of pulmonary colonization in the future. Studies using immunosuppressed animals suggest a correlation of *Pneumocystis*-DNA results between non-invasive samples like nasopharyngeal aspirates and lung tissue samples [19–21]. However, in human adults, a single diagnostic nasopharyngeal aspirate sample may detect only a fraction of *Pneumocystis* upper airway carriers, and sampling the upper airways more than once may increase the diagnostic yield for *Pneumocystis* [22, 23]. This means that a single upper airway specimen in adults is not necessarily representative of lung infection. By contrast, it has been documented that *Pneumocystis*-DNA in non-invasive samples from non-immunocompromised humans may represent nasal carriage acquired from an external source of contagion, indicating either transient or long-term colonization in the upper airways [23]. Studies that compare the yield of nasopharyngeal aspirate samples, bronchoalveolar lavage samples, and lung samples in the same individual patients will be difficult to perform and are lacking. Nasopharyngeal aspirate samples in healthy immunocompetent adults have an approximate yield of 20% [24]. A study of bronchoalveolar lavage samples in immunocompetent individuals undergoing bronchoscopy as part of a diagnostic work-up for possible respiratory disease in the United Kingdom, documented that 18% were colonized with *P. jirovecii* [25]. The yield of lung tissue samples is higher as documented in immunocompetent adults in Chile and Turkey, and detection rate varied depending on sampling procedures. They documented an average of 65% *P. jirovecii*-positive cases in a Chilean series after analysis of a median of seven specimens of 0.5 g each per lung to amount to 7% of total lung weight, and an average of 18.5% of *P. jirovecii*-positive cases in the series from Turkey after analysis of 1 g of lung tissue from a single specimen. This suggests that, similar as in non-invasive sampling [22], the study of multiple autopsy lung samples increases the yield and is therefore necessary to recognize the prevalence of the mild and focal pulmonary infection by *Pneumocystis* in the non-immunosuppressed adult.

Future autopsy studies in non-immunosuppressed adults should continue to specify the mode or diagnosis of death, and include the number of pulmonary samples analyzed, their total weight, site of origin, precautions to avoid contamination, DNA extraction method, and gene target amplified, as essential information for comparison and reproducibility of results. Future studies in autopsied infant lungs should disclose infant ages in addition to the above.

## References

[1] Miller RF, Huang L, Walzer PD (2013). *Pneumocystis* pneumonia associated with human immunodeficiency virus. Clin Chest Med.

[2] Ponce CA, Gallo M, Bustamante R, Vargas SL (2010). *Pneumocystis* colonization is highly prevalent in the autopsied lungs of the general population. Clin Infect Dis.

[3] Özkoç S, Köker M, Önder M, Delibaş SB (2016). Prevalence of *Pneumocystis jirovecii* colonization in autopsy cases in Turkey. J Med Microbiol.

[4] Beard CB, Fox MR, Lawrence GG, Guarner J, Hanzlick RL, Huang L (2005). Genetic differences in *Pneumocystis* isolates recovered from immunocompetent infants and from adults with AIDS: epidemiological implications. J Infect Dis.

[5] Vargas SL, Ponce CA, Hughes WT, Wakefield AE, Weitz JC, Donoso S (1999). Association of primary *Pneumocystis carinii* infection and sudden infant death syndrome. Clin Infect Dis.

[6] Vargas SL, Ponce CA, Gallo M, Perez F, Astorga JF, Bustamante R (2013). Near-universal prevalence of *Pneumocystis* and associated increase in mucus in the lungs of infants with sudden unexpected death. Clin Infect Dis.

[7] Sivam S, Sciurba FC, Lucht LA, Zhang Y, Duncan SR, Norris KA (2011). Distribution of *Pneumocystis jirovecii* in lungs from colonized COPD patients. Diagn Microbiol Infect Dis.

[8] Eddens T, Campfield BT, Serody K, Manni ML, Horne W, Elsegeiny W (2016). A novel CD4+ T cell-dependent murine model of *Pneumocystis*-driven asthma-like pathology. Am J Respir Crit Care Med.

[9] Huggett JF, Taylor MS, Kocjan G, Evans HE, Morris-Jones S, Gant V (2008). Development and evaluation of a real-time PCR assay for detection of *Pneumocystis jirovecii* DNA in bronchoalveolar lavage fluid of HIV-infected patients. Thorax.

[10] Ruiz-Fuentes JL, Diaz A, Entenza AE, Frion Y, Suarez O, Torres P (2015). Comparison of four DNA extraction methods for the detection of *Mycobacterium leprae* from Ziehl-Neelsen-stained microscopic slides. Int J Mycobacteriol.

[11] Helweg-Larsen J, Jensen JS, Dohn B, Benfield TL, Lundgren B (2002). Detection of *Pneumocystis* DNA in samples from patients suspected of bacterial pneumonia—a case-control study. BMC Infect Dis.

[12] Damiani C, Le Gal S, Da Costa C, Virmaux M, Nevez G, Totet A (2013). Combined quantification of pulmonary *Pneumocystis jirovecii* DNA and serum (1→3)-β-d-glucan for differential diagnosis of *Pneumocystis* pneumonia and *Pneumocystis* colonization. J Clin Microbiol.

[13] Vargas SL, Ponce CA, Galvez P, Ibarra C, Haas EA, Chadwick AE (2007). *Pneumocystis* is not a direct cause of sudden infant death syndrome. Pediatr Infect Dis J.

[14] Vargas SL, Ponce CA, Luchsinger V, Silva C, Gallo M, Lopez R (2005). Detection of *Pneumocystis carinii* f. sp. hominis and viruses in presumably immunocompetent infants who died in the hospital or in the community. J Infect Dis.

[15] Larsen HH, von Linstow ML, Lundgren B, Hogh B, Westh H, Lundgren JD (2007). Primary *Pneumocystis* infection in infants hospitalized with acute respiratory tract infection. Emerg Infect Dis.

[16] Ambrose HE, Ponce CA, Wakefield AE, Miller RF, Vargas SL (2001). Distribution of *Pneumocystis carinii* f. sp. hominis types in the lung of a child dying of *Pneumocystis* pneumonia. Clin Infect Dis.

[17] Helweg-Larsen J, Lundgren B, Lundgren JD (2001). Heterogeneity and compartmentalization of *Pneumocystis carinii* f. sp. hominis genotypes in autopsy lungs. J Clin Microbiol.

[18] Peters SE, Wakefield AE, Sinclair K, Millard PR, Hopkin JM (1992). A search for *Pneumocystis carinii* in post-mortem lungs by DNA amplification. J Pathol.

[19] Choukri F, Aliouat el M, Menotti J, Totet A, Gantois N, Garin YJ (2011). Dynamics of *Pneumocystis carinii* air shedding during experimental pneumocystosis. J Infect Dis.

[20] Linke MJ, Rebholz S, Collins M, Tanaka R, Cushion MT (2003). Noninvasive method for monitoring *Pneumocystis carinii* pneumonia. Emerg Infect Dis.

[21] Menotti J, Emmanuel A, Bouchekouk C, Chabe M, Choukri F, Pottier M (2013). Evidence of airborne excretion of *Pneumocystis carinii* during infection in immunocompetent rats. Lung involvement and antibody response. PLoS One.

[22] Vargas SL, Pizarro P, Lopez-Vieyra M, Neira-Aviles P, Bustamante R, Ponce CA (2010). *Pneumocystis* colonization in older adults and diagnostic yield of single versus paired noninvasive respiratory sampling. Clin Infect Dis.

[23] Vargas SL, Ponce CA, Gigliotti F, Ulloa AV, Prieto S, Munoz MP et al (2000) Transmission of *Pneumocystis carinii* DNA from a patient with P. *carinii* pneumonia to immunocompetent contact health care workers. J Clin Microbiol 38(4):1536–1538 10.1128/jcm.38.4.1536-1538.2000 PMC86483 10747139

[24] Medrano FJ, Montes-Cano M, Conde M, de la Horra C, Respaldiza N, Gasch A (2005). *Pneumocystis jiroveciii* in general population. Emerg Infect Dis.

[25] Maskell NA, Waine DJ, Lindley A, Pepperell JC, Wakefield AE, Miller RF (2003). Asymptomatic carriage of *Pneumocystis jiroveci* in subjects undergoing bronchoscopy: a prospective study. Thorax.

